# ER stress: an emerging regulator in GVHD development

**DOI:** 10.3389/fimmu.2023.1212215

**Published:** 2023-09-06

**Authors:** Hee-Jin Choi, Xue-Zhong Yu

**Affiliations:** Department of Microbiology & Immunology, Department of Medicine, and the Cancer Center, Medical College of Wisconsin, Milwaukee, WI, United States

**Keywords:** ER stress, unfolded protein response (UPR), GVHD, IRE-1, PERK, ATF6

## Abstract

Allogeneic hematopoietic cell transplantation (allo-HCT) is a promising therapeutic option for hematologic malignancies. However, the clinical benefits of allo-HCT are limited by the development of complications including graft-versus-host disease (GVHD). Conditioning regimens, such as chemotherapy and irradiation, which are administered to the patients prior to allo-HCT, can disrupt the endoplasmic reticulum (ER) homeostasis, and induce ER stress in the recipient’s cells. The conditioning regimen activates antigen-presenting cells (APCs), which, in turn, activate donor cells, leading to ER stress in the transplanted cells. The unfolded protein response (UPR) is an evolutionarily conserved signaling pathway that manages ER stress in response to cellular stress. UPR has been identified as a significant regulatory player that influences the function of various immune cells, including T cells, B cells, macrophages, and dendritic cells (DCs), in various disease progressions. Therefore, targeting the UPR pathway has garnered significant attention as a promising approach for the treatment of numerous diseases, such as cancer, neurodegeneration, diabetes, and inflammatory diseases. In this review, we summarize the current literature regarding the contribution of ER stress response to the development of GVHD in both hematopoietic and non-hematopoietic cells. Additionally, we explore the potential therapeutic implications of targeting UPR to enhance the effectiveness of allo-HCT for patients with hematopoietic malignancies.

## 1 Introduction

The endoplasmic reticulum (ER) is one of the largest organelles found within the cells of eukaryotic organisms and is the main place for the synthesis, folding, and modification of secretory or transmembrane proteins (1, 2). However, various conditions including hypoxia, oxidative stress, infection, or cancer might hamper the ER homeostasis and induce the ER stress (3). Cells activate a conserved adaptive signaling pathway, known as the unfolded protein response (UPR), to manage ER stress and control protein quality (4). Growing evidence indicates that UPR plays a significant role in the development and progression of various diseases including cancer, diabetes, neurodegenerative disorders, autoimmune diseases, and inflammatory (5–7). In this review, we focus on the regulation of the homeostasis and activation of hematopoietic and non-hematopoietic cells by the ER stress response and its potential impact on the development of GVHD.

## 2 Molecular mechanism of ER stress response

There are three primary UPR mediators including inositol-requiring enzyme-1 (IRE-1), protein kinase R (PKR)-like endoplasmic reticulum kinase (PERK), and activating transcription factor 6 (ATF6) (8). In the rest status, chaperon protein Bip binds to the ER luminal domain of three mediators, preventing their activation and keeping them in a monomeric, inactive state. However, upon accumulation of misfolded proteins within the ER lumen, Bip dissociates from PERK, IRE-1, and ATF6, triggering their activation (9–11).

### 2.1 IRE-1

IRE-1 has two isoforms, namely IRE-1α and IRE-1β (12). IRE-1α has a broader expression pattern across tissues and cell types than IRE-1β, and it plays a major role in the UPR (13). The cytoplasmic region of IRE-1α contains both Ser/Thr kinase and endoribonuclease (RNase) domains (14). IRE-1α activation occurs through oligomerization and trans-autophosphorylation, which induces a structural change, ultimately activating the RNase domain (15). Upon activation, IRE-1α cleaves a 26-nucleotide intron from the mRNA encoding the transcription factor X-box binding protein-1 (XBP-1). This alteration results in expression of a more stable and active form, referred to as XBP-1s (6). Upon binding to the UPR element (UPRE) that contains the consensus sequence TGACGTGG/A, XBP-1s functions as a transcription factor for UPR-related genes (15). In addition, IRE-1α possesses the distinctive capability of inducing IRE1-dependent decay (RIDD) of mRNA, which helps to alleviate a stressed ER from the pressure of newly synthesized proteins (16). Besides its nucleolytic activity, IRE-1α also possesses signaling functions. For instance, it activates the stress-induced Jun N-terminal kinase (JNK)2 and interacts with components of the cell-death machinery, such as caspase-12 (17, 18). Increasing evidence demonstrates that non-coding RNAs, including microRNAs (miRNAs), play significant roles in the ER stress response. The activity of IRE-1α is commonly linked to the suppression of miRNA accumulation due to its RNase function. Several miRNAs including miRs-17, 34a, 96, and 125b were shown to repress the translation of pro-apoptotic proteins (19, 20).

### 2.2 PERK

PERK plays a dual role, able to detect the magnitude of ER stress and initiate growth arrest for repair, or activate apoptosis when ER stress becomes too severe (9). Upon activation, PERK undergoes oligomerization and autophosphorylation in its cytosolic kinase domain (8). Subsequently, PERK phosphorylates the eukaryotic translation initiation factor 2 (eIF2α), leading to the inhibition of mRNA translation (15). On the other hand, specific mRNAs that contain short open reading frames in their untranslated regions can still be translated when eIF2 is phosphorylated. Among these mRNAs is one that encodes the transcription factor ATF4 (21). The C/EBP Homologous Protein (CHOP) and growth arrest and DNA damage–inducible 34 (GADD34) are two significant target genes that are regulated by ATF4 (21). If ER stress persists, CHOP activates the genes responsible for encoding death receptor 5 (DR5) and Bim, ultimately leading to apoptosis (22, 23). GADD34 functions as a negative feedback mechanism by encoding a protein phosphatase 1 (PP1) that counteracts PERK by dephosphorylating eIF2α (24). The PERK signaling pathway also triggers the phosphorylation and subsequent activation of the other transcription factor known as nuclear factor erythroid 2-related factor 2 (NRF2) (25). PERK can trigger the induction of various miRNAs including miR-211/204, miR-708, miR-483 and miR-216b (26–29). However, the implications of PERK-related activities on miRNA are contentious. While some of miRNAs induced by PERK are associated with pro-survival functions (26), the others, such as with miR-216b miR-483 and miR-211-5p, may promote apoptosis (27–29). Conversely, PERK can be directly targeted by certain miRNAs like miR-204 and miR-1283, which, as a result, suppress PERK signaling (30, 31).

### 2.3 ATF6

ATF6 consists of a bZIP domain located in the cytosol and a stress-sensing domain situated within the ER lumen (15). Upon ER stress condition, ATF6 is enclosed within transport vesicles that detach from the ER and carry it to the Golgi apparatus, where site-1 (S1P) and site-2 (S2P) proteases cleave ATF6 at two distinct sites in a sequential manner (32). Subsequently, the released N-terminal cytosolic segment of ATF6, known as ATF6(N), translocate to the nucleus and triggers the activation of genes targeted by the UPR (21). ATF6 also modifies the levels of various miRNAs, such as miR-721, miR-363, and miR-455, with some of these miRNAs being downregulated, which could contribute to the increase of their corresponding mRNAs (33).

## 3 An overview of GVHD pathogenesis

The classification of GVHD as acute or chronic has traditionally been based on the timing of its presentation, with a cutoff of 100 days after transplantation (34). The latest classification from the National Institutes of Health (NIH) includes the identification of late-onset acute GVHD (occurring after day 100) and an overlap syndrome that exhibits characteristics of both acute and chronic GVHD (35). The 2014 NIH Consensus Conference revised the diagnostic and scoring criteria for cGVHD to provide a more accurate measurement of the disease burden and facilitate further research (36). The update clarified the definition of overlap chronic GVHD, modified diagnostic criteria for organ system involvement, revised organ-specific severity score, removed certain indicators, and incorporated abnormalities not due to GVHD into the organ-specific scores (36).

### 3.1 Pathogenesis of acute GVHD

Acute GVHD (aGVHD) develops as a result of three underlying processes (37). First, the administration of chemotherapy and radiotherapy during the conditioning phase prior to graft infusion can cause tissue damage and result in the release of various molecular activators of the immune response that can enhance the presentation of alloantigen by antigen-presenting cells (APCs) in the host (38). Subsequently, in the early post-HCT phase, donor T cells interact with host APCs, followed by donor APCs later on. These interactions provide costimulatory signals that activate and differentiate donor T cells into subtypes including T-helper (Th) 1, T-cytotoxic (Tc) 1, and Th17/Tc17 (39). In the final step, the cytotoxic effector T cells and phagocytes migrate towards target organs and release inflammatory cytokines including IFN-γ, TNFα, IL-1β, IL-2, and IL-17, resulting in tissue damage (40).

### 3.2 Pathogenesis of chronic GVHD

The pathogenesis of chronic GVHD (cGVHD) also can be characterized by three phases (41). The early phase of cGVHD is marked by the rapid stimulation of non-hematopoietic cells, such as fibroblasts and endothelial cells, along with innate immune cells. In this stage, T cells become activated upon encountering APCs that increase the expression of co-stimulatory molecules due to tissue damage related to conditioning (42). The involvement of pathogenic Th17 cells has been suggested, as suppressing IL-17-producing cells through programmed death 1 (PD-1) or IL-12 blockade leads to a reduction in cGVHD symptoms in the skin, liver, and salivary glands (43, 44). Phase II is characterized by the adaptive activation of effector cells in the immune system, especially T and B cells (41). Somatic hypermutation in B cells leads to the production of autoreactive antibodies, which can contribute to the development of cGVHD in the skin, bronchiolitis obliterans, and liver damage (45). A crucial element in the development of cGVHD is the damage to the thymus caused by alloreactive T cells, resulting in the loss of the central tolerance loss and release of autoreactive CD4+ T cells (46). The development of cGVHD is facilitated by a lack of regulatory T cells (Tregs), regulatory B cells (Bregs), and CD4+ iNKT cells (41). Abnormal tissue repair and activation of fibroblasts are characteristic features of phase 3 (41). The production of extracellular matrix collagen and biglycan by activated fibroblasts leads to the cross-linking of collagen, resulting in an elevation of tissue stiffness (47).

## 4 The contribution of UPR in GVHD development

The underlying mechanism of GVHD is the recognition of the host major or minor histocompatibility antigens by T cells. While the T cells from the donor are the primary cause of GVHD, both the donor and host APCs can exhibit host antigens to the donor T cells (40). The induction of GVHD depends mainly on host APCs, but donor APCs could enhance this effect (48, 49). As a result of HCT conditioning and GVHD, MHC expression is induced on the endothelial and epithelial cells of target tissues, resulting in the acquisition of functional antigen-presenting activity (50, 51). Hence, it is possible for both hematopoietic and non-hematopoietic APCs in the host to trigger GVHD. Other than the immune system, the skin, liver, lung and intestine are the main organs that can be affected in GVHD (52). Due to the presence of numerous highly secretory cells, ER stress and UPR have significant roles in various biological processes and are involved in the pathogenesis of these tissues (53, 54). Therefore, we outline below how the UPR impacts non-hematopoietic cells as well as regulates hematopoietic immune cells in the development of GVHD.

### 4.1 Role of UPR in hematopoietic cells

#### 4.1.1 Dendritic cells

The induction of acute GVHD requires hematopoietic APCs, and due to their efficiency, DCs are considered the most important APC population (40, 48). Conventional dendritic cells (cDCs) are the predominant DC subset, and they can be further divided into cDC1 and cDC2 types (3). cDC1s express CD8α and have a specialized ability to cross-present exogenous antigens to CD8+ T cells while cDC2s express CD11b and have a critical role in activating CD4+ T cells (3). Plasmacytoid dendritic cells (pDCs) make up a minor population of DCs, but they have a unique ability to sense nucleic acids from pathogens and produce type I interferon (55). One of the initial studies demonstrated the association between ER stress and DCs and revealed a significant impairment in the development and survival of DCs upon XBP-1 deletion in the hematopoietic compartment (56). The authors reported that pDCs exhibited notably higher levels of XBP-1 splicing compared to cDCs and were more adversely affected by XBP-1 deficiency (56). A more recent study, however, demonstrated that cDCs exhibit the highest IRE1α activity in the ER stress-activated indicator (ERAI) mouse model (57). Moreover, the deletion of XBP-1 in cDC1s disrupted antigen presentation by directly modulating the expression of key proteins involved in loading antigens onto the MHC complex (57, 58). Interestingly, the loss of XBP1 in mucosal cDC1s affects tissue-specific survival differently; specifically, lung cDC1s die while intestinal cDC1s can survive (59). These results imply that the threshold for IRE1 activation varies across different mouse strain, environment, tissues, and DC subtypes.

**Figure 1:**
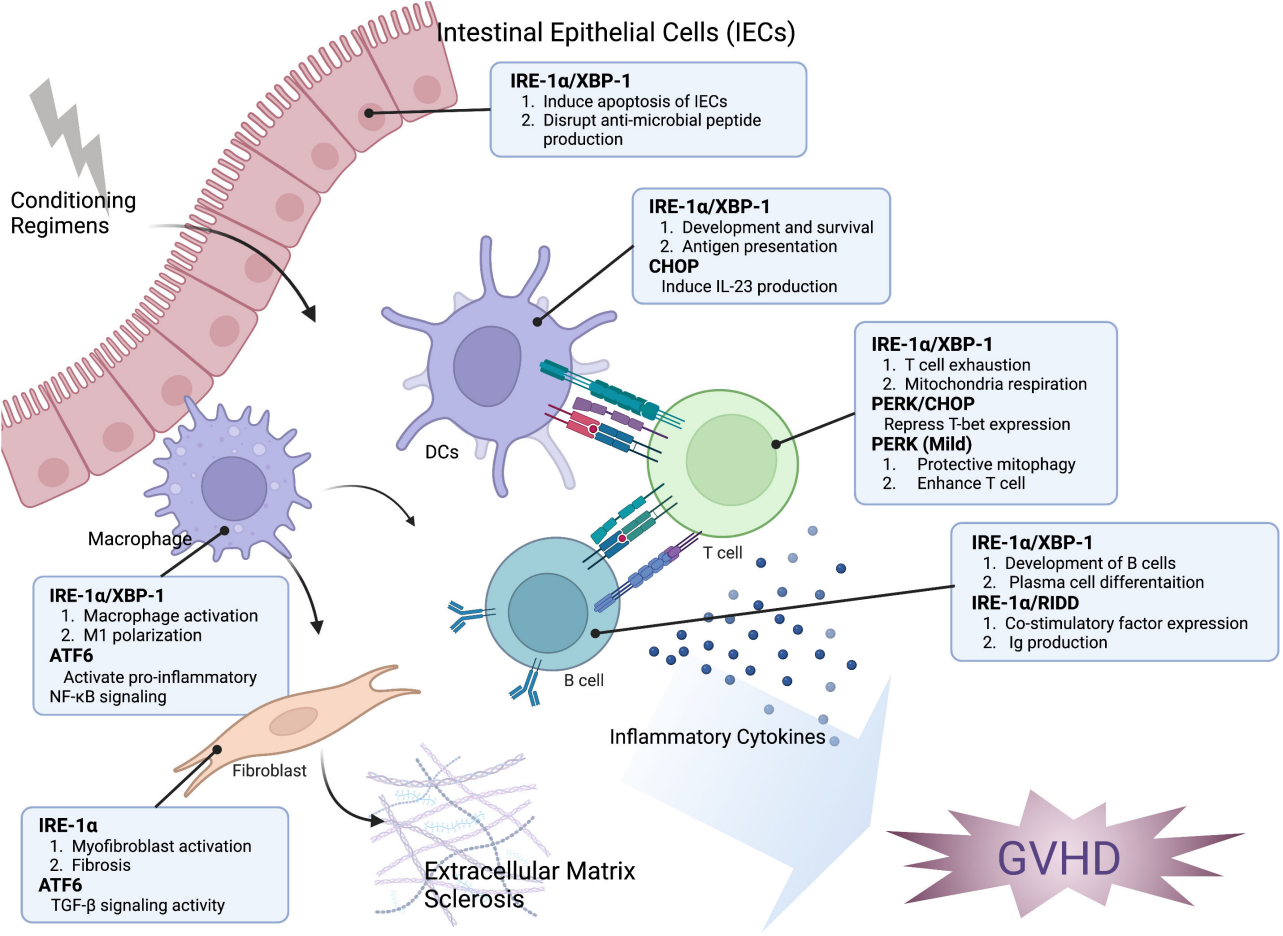
The impact of the ER stress response on the development of GVHD. The modulation of hematopoietic and non-hematopoietic cell function in the development of GVHD is significantly influenced by ER stress response. XBP-1 can interfere with the generation of antimicrobial peptides, change the microbiome composition, and trigger the apoptosis of intestinal epithelial cells (IECs). The IRE-1α/XBP-1 signaling pathway is crucial for both the development and antigen-presenting ability of dendritic cells (DCs). Additionally, CHOP plays a role in IL-23 production by DCs, which is important for Th17 differentiation in the development of GVHD. The activation of IRE-1 and ATF6 is related to the polarization and pro-inflammatory activity of macrophages. IRE-1 activation can induce T cell exhaustion and repress mitochondrial respiration, while PERK activation can reduce T-bet expression and subsequently decrease Th1 activation. However, mild activation of PERK can induce protective mitophagy and enhance T cell function. The induction of RIDD through XBP-1 inhibition resulted in a decrease of B cell pathogenicity. Activation of myofibroblast, which leads to accelerated fibrosis, is associated with the activation of IRE-1 and ATF6. Taken together, targeting the ER stress response may be an effective approach for controlling GVHD and malignant relapse.

In the presence of ER stress, treatment with toll-like receptor (TLR) agonists results in a substantial increase in CHOP activation and its direct binding to the promoter region of the pro-inflammatory cytokine IL-23p19 in DCs (60). Expanding and maintaining the Th17 population, crucial for the development of acute and chronic GVHD, is significantly influenced by IL-23 (61). The activation of STAT3 signaling by IL-23 leads to an increase in the expression of RORγt, the key transcription factor of Th17 cells (62). In GVHD, the targeted deletion of XBP-1s in DCs effectively inhibits alloreactive CD4 T cells and the pharmacological inhibition of XBP-1 offers protection against GVHD (63). The authors also presented evidence that this phenomenon is specific to GVHD since the T cell response within the tumor was not impacted by XBP-1 deficient DCs (63). Thus, ER stress may serve as a critical mediator for regulating DCs functions by controlling antigen presentation and cytokine production (**Figure 1**). However, the impact on GVHD through specifically targeting XBP-1 in DCs remains to be investigated. Additional research is required to gain a comprehensive understanding of how UPR distinguishes between the alloreactive response and antitumor activity.

#### 4.1.2 Macrophages

Macrophages become primed due to the allogeneic reaction and production of inflammatory cytokines and chemokines, which lead to the characteristic symptoms of acute GVHD (39, 64). Moreover, activated macrophages that produce TGF-β and platelet-derived growth factor α (PDGF-α) promote aberrant tissue repair, resulting in the activation of fibroblasts in cGVHD (41). Both TLRs and NOD1/NOD2 receptors directly activate IRE-1/XBP-1 signaling in macrophages, enabling them to achieve a pro-inflammatory response (65, 66). In addition, IRE-1α regulates mitochondrial dysfunction via nucleotide-binding domain and leucine-rich repeat containing (NLRP3)-caspase-2, resulting in inflammasome activation and IL-1β production (67). Macrophage polarization was also mediated by IRE-1α, with promotion of M2 polarization and decrease in M1 polarization observed upon abrogation of IRE-1α (68). In GVHD, the polarization of M1 macrophages mediated by the NLRP3 inflammasome drives the differentiation of Th1 and Th17 cells, leading to the disease progression (69). The role of ATF6 in macrophages was demonstrated to regulate the TLR-mediated pro-inflammatory response in ER-stressed macrophages by activating NF-κB and limiting Akt activation (70). These findings prove that ER stress response plays an active role in the pathophysiological mechanisms of macrophages by regulating cytokine expression and polarization (**Figure 1**). Therefore, while the direct impact of UPR in macrophages on GVHD development is not yet clear, it is possible that inhibiting UPR in macrophages could potentially provide benefits for GVHD prevention. Additional studies are necessary to determine how the UPR contributes to the development of GVHD through regulating macrophages.

#### 4.1.3 T cells

Donor T cells that react against host tissues are primarily responsible for causing GVHD (37). Moreover, the differentiation of CD4 T cells into different lineages of Th cells, including Th1 and Th17 cells, is closely associated with the development of GVHD (37). Previous studies have reported that the regulation of Th2 and Th17 differentiation is mediated by eIF2α and XBP-1 signaling pathways, respectively (71). In addition, activation of the IRE1-XBP1 pathway was observed during acute infection and found to be crucial for the differentiation of CD8 T cells into effector T cells (72). However, the role of the UPR in the activation of anti-tumoral T cells remains a topic of controversy. In certain tumor models, tumor cells triggered the UPR to impede the anti-tumor activity of T cells (73–75). Elevated CHOP in tumor infiltrated CD8 T cells directly cause repression of T-bet expression, a master regulator of effector T cell function (73). The accumulation of cholesterol in tumor-infiltrating CD8 T cells induced XBP-1 signaling, which was linked to increased expression of immune checkpoints and functional exhaustion of these T cells (75). In ovarian cancer, IRE-1α-XBP-1 signaling pathway plays a crucial role in regulating T cell function, including mitochondrial activity (74). It has been demonstrated that XBP-1 regulates the number of glutamine carriers, which in turn restricts the inflow of glutamine required to maintain T cell mitochondrial respiration under glucose-deprived conditions (74). On the contrary, a moderate activation of PERK by carbon monoxide led to the suspension of protein translation, induced protective mitophagy and enhanced mitochondrial function, ultimately boosting the efficacy of anti-tumor T cells (76). In general, the function of UPR in T-cell allogeneic response remains largely unknown. As the UPR can enhance or suppress T cell function depending on the intensity of ER stress and the microenvironment (**Figure 1**), additional research is required to reveal the role of UPR in T-cell pathogenicity in the development of GVHD.

#### 4.1.4 B cells

The role of B cells in controlling the pathogenesis of aGVHD remains controversial (77, 78). However, in the case of cGVHD, there is a significant body of evidence suggesting an interplay between donor T and B cells in disease pathogenesis (47). Studies have shown that the IRE-1α/XBP-1s signaling pathway plays a crucial role in both the development of B cells and the differentiation of plasma cells, which are responsible for producing high levels of immunoglobulin (Ig) (79, 80). We have shown that deletion of XBP-1 specifically in B cells reduces the severity of cGVHD (81). This reduction is associated with impaired B-cell functions, including decreased expression of co-stimulatory factors, reduced Ig production, and impaired differentiation of germinal center cells, as well as reduced T-cell responses (81). Interestingly, the reduced pathogenicity of XBP-1-deficient B cells in cGVHD can be reversed by restricting RIDD activity (82). Furthermore, restraining RIDD activity in B cells alone led to increased severity of chronic GVHD (82). These findings indicate that RIDD is an important mediator in reducing the pathogenesis of cGVHD by targeting XBP-1s (82) (**Figure 1**). While the significance of IgG has been reported, the impact of IgM, a notable target of RIDD in B cells, on GVHD is not well understood (83, 84). Therefore, additional research is necessary to ascertain the role of RIDD-mediated IgM in B cells and its influence on GVHD development. In addition, despite B cells being able to activate all three branches of the UPR in response to pharmacological inducers, there is limited research on the role of PERK and ATF6 in B cells and their contribution to the development of GVHD (85), which deserves further studies.

### 4.2 Role of UPR in non-hematopoietic cells

#### 4.2.1 Intestinal epithelial cells

The intestinal epithelium serves as a vital intermediary and barrier between the luminal environment and the immune system of the host due to the presence of numerous highly secretory cells (54). Intestinal epithelial cells (IECs) are persistently exposed to microbiota, toxins, and metabolites, which lead to the production of a significant quantity of cytokines and other proteins, causing ER stress (86). Intestinal inflammation is primarily linked to the IRE-1α-XBP1 branch of the UPR pathway, as evidenced by the heightened susceptibility of induced colitis in IECs deficient for IRE-1α or XBP-1 (7, 13). On the other hand, in the context of GVHD, ER stress is induced in intestinal cells, which can disrupt the production of anti-microbial peptides, alter the composition of the microbiome, and affect the activity of pro-apoptotic pathways (87). Therefore, either genetic or pharmacological blockage of IRE-1/XBP-1 signaling can reduce the severity of GVHD by directly protecting the intestinal epithelium (87) (**Figure 1**). Additional research is required to determine the underlying mechanisms by which IRE-1/XBP-1 regulates gut epithelium differentially in GVHD and colitis models.

#### 4.2.2 Fibroblast

The activation of myofibroblasts leads to excessive production and deposition of collagen and other extracellular matrix proteins, which characterizes fibrosis and is a significant pathological feature of cGVHD (41, 88). A prior investigation demonstrated that through the cleavage of miR-150, IRE-1α can stimulate myofibroblast activation and trigger fibrosis (20). The pharmacological inhibition of IRE-1α decreased skin fibrosis and reversed the fibrotic phenotype of myofibroblasts that were extracted from patients with scleroderma (20). A recent study demonstrated that ATF6 transcriptionally controls the expression of thioredoxin domain-containing protein 5 (TXNDC5), which promotes fibrosis by enhancing TGF-β signaling activity in kidney fibroblasts (89). ER stress can activate fibroblasts and promote pulmonary fibrosis through a positive feedback loop with ZC3H4, a recently discovered member of the CCCH-type zinc finger protein family (90). These data suggest that ER stress plays a crucial role in fibroblast activation and could be a potential target for regulating fibrosis in cGVHD (**Figure 1**). However, further research is necessary to validate this hypothesis.

## 5 Conclusion

GVHD control is a delicate task, as suppressing T cell response to prevent GVHD may also compromise GVL activity and potentially lead to tumor relapse. UPR can have a dual effect on the regulation of immune cell activity, which depends on the severity of ER stress. Therefore, we propose that regulating UPR may be a viable strategy for GVHD prevention while preserving the GVL activity. It is also possible that ER stress may serve as potential biomarkers to predict GVHD onset, in that elevated expressions of GRP78 and CHOP in patient biopsies were shown to correlate with the severity of GVHD (87). The IRE-1α-XBP-1 signaling pathway appears to be a promising target for GVHD, as inhibiting this pathway in DCs, B cells, and IECs has been shown to reduce their function and the severity of GVHD. However, XBP-1 inhibition has been found to increase the activity of T cells, providing a rationale in preserving the GVL effect upon XBP-1 inhibition in HCT. The impact of PERK on T cell activity is disputed, as PERK can either decrease or increase T cell activity depending on the levels of PERK induction. Based on our findings, we propose that targeting the IRE-1α and PERK signaling pathways could serve as potential strategies to mitigate GVHD. Specifically, IRE-1α plays a critical role in the function of DCs by stimulating T cells and increasing damage to IECs. Thus, IRE-1 may represent a promising target for cGVHD due to its significant involvement in activating B cells, promoting fibroblast activation, and facilitating M2 macrophage polarization. On the other hand, PERK is involved in T cell activation, which is an essential pathogenic process in the development of aGVHD. In addition, the role of ATF6 in GVHD progression remains largely unexplored due to the lack of efficient tools for detecting pathway activation. The three branches of the ER stress response can interact with one another, and therefore regulating these interactions could be a potential strategy for distinguishing GVH and GVL responses. Further research is necessary to comprehend the influence of PERK and ATF6 signaling on disease progression and to investigate strategies for regulating the interplay of the three UPR mediators, aiming to prevent GVHD while maintaining GVL activity in allo-HCT.

## Author contributions

H-JC wrote the manuscript. X-ZY edited and revised the manuscript. All authors contributed to the article and approved the submitted version.
